# Morphological and genetic analysis for the diversity conservation of rare species, *Thamnaconus multilineatus* (Tetraodontiformes: Monacanthidae)

**DOI:** 10.1371/journal.pone.0292916

**Published:** 2024-02-29

**Authors:** Tae-Sik Yu, Kiyun Park, Kyeong-Ho Han, Ihn-Sil Kwak

**Affiliations:** 1 Fisheries Science Institute, Chonnam National University, Yeosu, Republic of Korea; 2 Department of Aquaculture, Chonnam National University, Yeosu, Republic of Korea; 3 Department of Ocean Integrated Science, Chonnam National University, Yeosu, Republic of Korea; Università degli Studi di Torino, ITALY

## Abstract

Climate changes have altered biodiversity and ultimately induced community changes that have threatened the survival of certain aquatic organisms such as fish species. Obtaining biological and genetic information on endangered fish species is critical for ecological population management. *Thamnaconus multilineatus*, registered as an endangered species by the IUCN in 2019, is a Data Deficient (DD) species with a remarkably small number of habitats worldwide and no known information other than its habitat and external form. In this study, we characterized the external and osteological morphology of a *T*. *multilineatus* specimen collected from eastern Jeju Island, South Korea, in 2020. We also investigated the phylogenetic relationships among related fish species through complete mitochondrial DNA (mtDNA) analysis of the *T*. *multilineatus* specimen. The external and skeletal characteristics of *T*. *multilineatus* were similar to those of previous reports describing other fish of the genus *Thamnaconus*, making it difficult to classify *T*. *multilineatus* as a similar species based only on morphological characteristics. As a result of analyzing the complete mtDNA of *T*. *multilineatus*, the length of the mtDNA was determined to be 16,435 bp, and the mitochondrial genome was found to have 37 CDCs, including 13 PCGs, 22 tRNAs, and 2 rRNAs. In the phylogenetic analysis within the suborder Balistoidei, *T*. *multilineatus* mtDNA formed a cluster with fish of the genus *Thamnaconus*. This study is the first to report on the skeletal structure and complete mtDNA of *T*. *multilineatus*. Since the current research on *T*. *multilineatus* has only been reported on morphology, the results of this study will be utilized as important information for the management and restoration of *T*. *multilineatus* as an endangered species and significant fishery resource.

## Introduction

The striped filefish (*Thamnaconus multilineatus*) has been registered as a data deficient species on the 2019 International Union for Conservation of Nature (IUCN) Red List, the most comprehensive list of threatened species, with only data on its external morphology and sampling areas reported [1–4]. Essential data, including morphology, genetics, and ecological information, are necessary to manage endangered species. In particular, the higher taxonomic group *T*. *multilineatus*, of the family Monacanthidae, consists of many morphologically similar species [5]. Therefore, information about skeletal features, genetics, and the commonly utilized morphological characteristics is needed for classification.

Numerous morphological, anatomical, and genetic studies of the family Monacanthidae have been conducted to resolve higher-level relationships [6–10]. Despite these previous studies, there remains difficulty in resolving higher-level taxonomic relationships. Classification systems that use morphological and anatomical characteristics face limitations, and the same is true for classification systems based on short sequences. Sequencing the complete mitochondrial genome is a method for determining higher-level relationships of complex taxa such as the family Monacanthidae [11]. The fish species of the family Monacanthidae for which complete mitochondrial DNA (mtDNA) have been reported include *Thamnaconus tessellatus*, *Thamnaconus hypargyreus*, *Thamnaconus modestus*, *Thamnaconus septentrionalis*, *Aluterus monoceros*, *Aluterus scriptus*, *Meuschenia hippocrepis*, and *Pseudalutarius nasicornis* [12–17].

The striped filefish has been difficult to classify among the genera *Pseudomonacanthus*, *Cantherhines*, and *Thamnaconus*. The striped filefish was first reported as *P*. *multilineatus* Tanaka, 1918. After this first report, various fish researchers classified *P*. *multilineatus* in the genus *Cantherhines* [18, 19]. Although Hutchins and Randall [20] mentioned that *C*. *multilineatus* should be classified in the genus *Thamnaconus*, they did not provide supportive evidence. *C*. *multilineatus* was later reported to belong to the genus *Cantherhines* in a review of Tetraodontiformes taxonomy and systematics [21]. This species is currently reported as *Thamnaconus multilineatus* [22], a greater understanding of its genetic and morphological characteristics are needed to better understand its phylogenetic position. In this study, we described the morphological and osteological characteristics of *T*. *multilineatus*. In addition, we sequenced the complete mtDNA genome, analyzed its genomic structure, and assessed the phylogenetic position of *T*. *multilineatus* within the family Monacanthidae for the first time. This basic information will contribute to the management of *T*. *multilineatus* and further phylogenetic studies of species in the family Monacanthidae.

## Materials and methods

### Ethical note statement

This research was undertaken in line with the ethical requirements of the Animal Care and Use Committee of Chonnam National University (Yeosu, Republic of Korea) with the approval code number: NUIACUC-YS-2019-6).

### Sampling site and identification

One specimen of *T*. *multilineatus* was sampled from eastern Jeju Island (33°26’48”N, 127°16’48”E) in October 2020 using a bottom trawl. The sample was frozen on-site and transported to the laboratory, where it was identified following Matsumoto et al. [23] and Peristiwady et al. [3].

### Observation of morphology

We photographed the sample to observe the external morphology, then counted the dorsal, pectoral, and anal fins, and measured each body part. Measurements were taken using a digital caliper to the nearest 0.1 mm. The standard length (SL) was measured from the upper lip to the base of the caudal fin. The body depth, head length, snout length, orbit diameter, predorsal length, preanal length, prepelvic length, caudal peduncle depth, dorsal-fin base, first dorsal spine, longest dorsal ray, anal-fin base, longest anal ray, caudal fin length, and pectoral-fin length were represented as a percentage of the standard length.

The specimen was then steamed, after which the muscles were removed to extract the skeleton and observe osteological characteristics. Each part of the extracted skeleton was photographed and sketched. The skeleton was divided into the following parts: cranium, vertebrae, opercular region, jaw bones, hyoid arch, branchiostegal rays, urohyal, mandibular region, and shoulder girdle. Osteological terminology follows Tyler [7].

### Characterization of the complete mitochondrial DNA sequence

Total genomic DNA was extracted from the fins of *T*. *multilineatus* using the DNeasy blood & tissue kit (Qiagen, Valencia, CA, USA). It was stored at Specimen Museum of Fisheries Science Institute, Chonnam National University (accession number CNUFSI-030123007). The library preparation and DNA sequencing (100 bp paired-end with different insert sizes; Illumina HiSeq4000) was carried out by Macrogen Inc. (Seoul, Korea). De novo assembly of cleaned reads was performed with various k-mer sizes using SPAdes v.3.13.0 [24]. After assembly, MitoZ (v.2.3), a Python3-based toolkit, was used for annotation [25].

### Phylogenetic analysis

The complete mitochondrial DNA sequences of 25 species of the family Monacanthidae have been reported in the National Center for Biotechnology Information (NCBI). The phylogenetic analysis was conducted to confirm the taxonomic position of *T*. *multilineatus* within the family Monacanthidae. Subsequently, a phylogenetic analysis was carried out with 18 species of the family Monacanthidae and 12 species of the family Balistidae belonging to the sister group using mtDNA COI DNA registered in the NCBI. The phylogenetic tree was constructed based on Neighbor-joining (NJ) in Mega X [26]. Bootstrap values are indicated at the nodes (1,000 replicates). The evolution model was established using jModelTest 2.1.10 [27], and the GTR+I+G (generalized time reversible with the invariable site and gamma distribution) model was used for NJ analysis.

## Results

### Morphological characteristics

*Thamnaconus multilineatus* (Tanaka, 1918)

(Fig 1A; Table 1)

**Fig 1:**
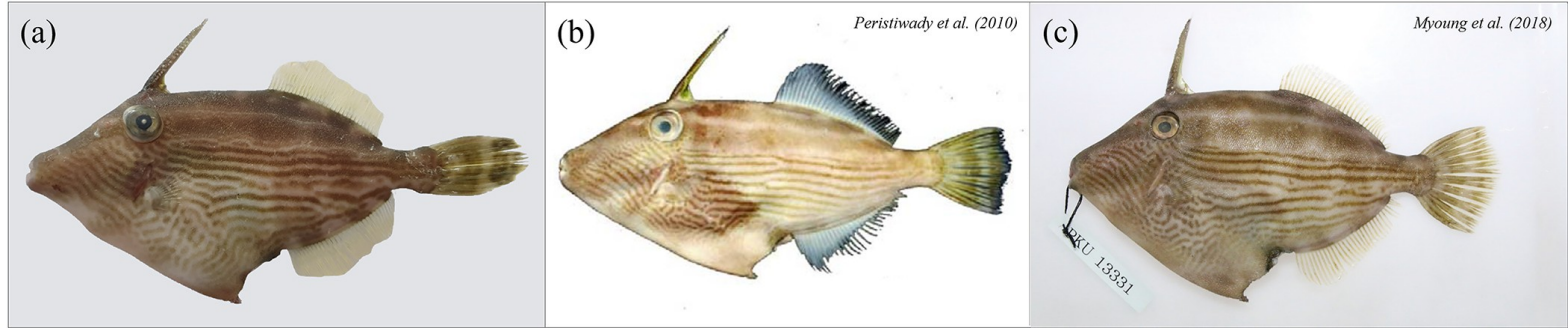
External view of *Thamanconus multilineatus*. (a) Specimen collected from Eastern Jeju island (present study, SL: 150.0 mm); (b) Specimen collected from Bitung, Indonesia (Peristiwady *et al*., 2010, SL: 192.0 mm); (c) Specimen collected from Northern Jeju island, Korea (Myoung *et al*., 2018, SL: 174.5 mm).

**Table 1:** Comparison of morphometric and meristic traits among *Thamnaconus multilineatus*.

Morphological characteristics	Present study	Myoung et al. [4]	Peristiwady et al. [3]
**Standard length (mm)**	150.0	174.5	191.5–192.0
As % SL			
Body depth	40.7	43.7	44.2
Head length	29.7	31.8	31.3
Snout length	25.8	28.5	27.3
Eye diameter	8.5	8.3	8.9
Predorsal length	29.9	34.3	32.8
Preanal length	60.3	67.4	62.6
Caudal-peduncle depth	8.6	8.4	8.0
Caudal-peduncle length	8.9	-	8.9
Dorsal fin base	32.6	33.1	30.8
Anal fin base	29.8	30.5	27.7
First dorsal spine	26.2	23.4	24.1
Longest soft dorsal ray	12.4	11.6	13.2
Longest anal ray	9.3	10.5	13.3
Pectoral fin length	8.6	9.7	10.6
Counts			
Dorsal fin rays	33	32	32–33
Pectoral fin rays	13	12	12–13
Anal fin rays	32	31	30–32

*Pseudomonacanthus multilineatus* Tanaka, 1918: 478 (type locality: Sagami Bay, Japan)

*Cantherhines multilineatus* (Tanaka, 1918): Kyushin *et al*., 1977: 356 (Japan) [18]; Masuda *et al*., 1984: 360 (Japan) [28]; Lindberg *et al*., 1997: 91 (Russia) [19]; Nakabo, 2000: 1405 (Japan) [29]; Nakabo, 2002: 1405 (Japan) [30]; Shinohara *et al*., 2005: 444 (Japan) [31]; Matsuura, 2015: 11 (Japan) [21]

#### Description

Counts and measurements of *T*. *multilineatus* are shown in Table 1. The body is compressed and oblong. The first dorsal spine is long and straight. The eyes are located below the first dorsal spine. Brown stripes are scattered across the lower 2/3^rds^ of the eyes. The color of the dorsal, pectoral, and anal fins is light brown, and spots are distributed on the caudal fin.

#### Distribution

*T*. *multilineatus* has been collected from Indonesia [3], Japan [23], Myanmar [32], and the Republic of Korea [4], and is distributed in the Indo-Pacific.

### Osteological description of *T*. *multilineatus*

**Fig 2:**
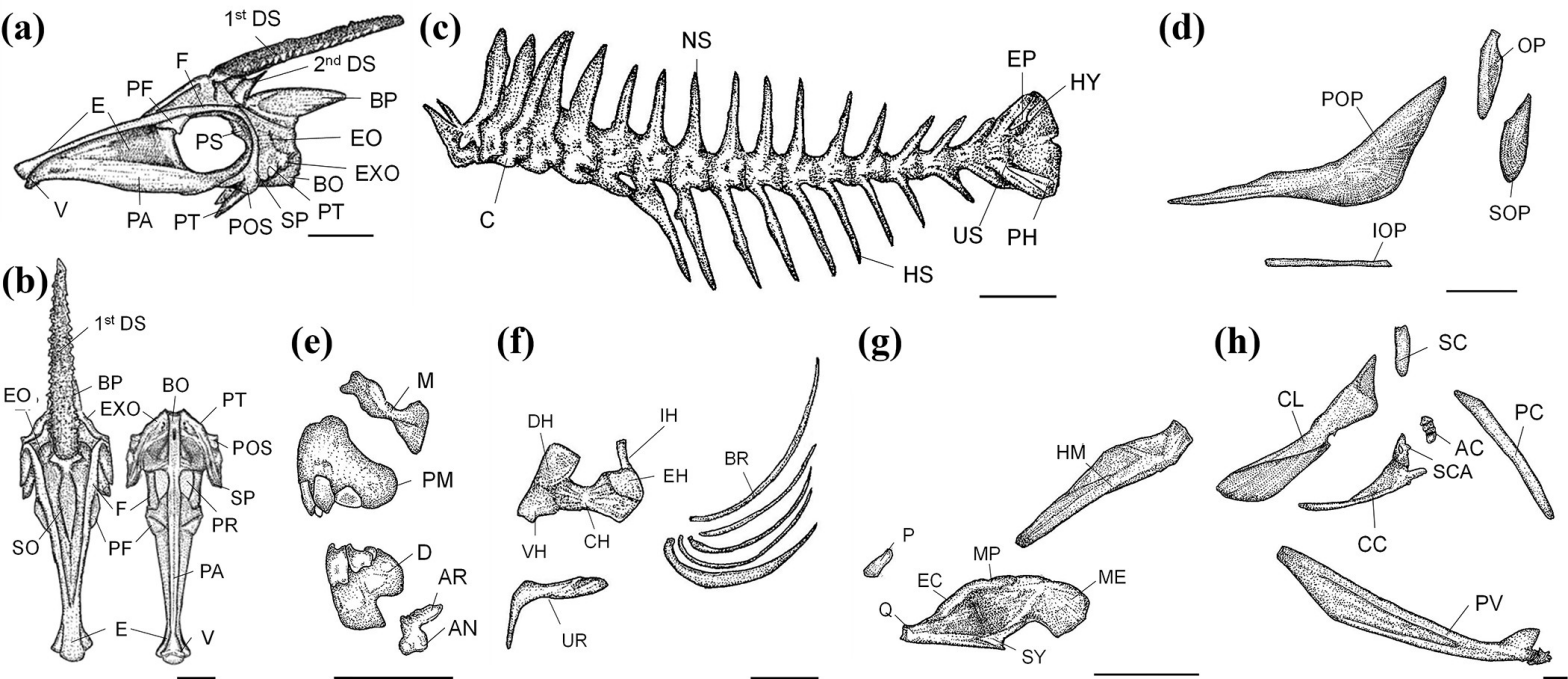
The osteological structure of *Thamnaconus multilineatus*. A: Lateral view of the cranium, B: Dorsal and ventral view of the cranium. C: Vertebrae, D: Opercular region, E: Jaw bones, F: Hyoid arch, branchiostegal rays, and urohyal, G: Mandibular region, H: Shoulder girdle. AN: Angular; AR: Articular; BO: Basioccipital; BP: Basal pterygiophore; BR: Branchiostegal rays; C: Centrum; CL: Cleithrum; CC: Coracoid; CH: Ceratohyal; D: Dentary; DH: Dorsal hypohyal; E: Ethmoid; EC: Ectopterygoid; EH: Epihyal; EO: Epiotic; EP: Epural; EXO: Exoccipital; F: Frontal; HM: Hyomandibular; HS: Hemal spine; HY: Hypural; IH: Interhyal; IOP: Interoperculum; M: Maxillary; ME: Metapterygoid; MP: Mesopterygoid; NS: Neural spine; OP: Operculum; P: Palatine; PA: Parasphenoid; PC: Postcleithra; PF: Prefrontal; PM: Premaxillary; POP: Preoperculum; POS: Posttemporal; PH: Parhypural; PR: Prootic; PT: Pterotic; PV: Pelvis; Q: Quadrate; SC: Supracleithrum; SCA: Scapula; SO: Supraoccipital; SOP: Suboperculum; SP: Sphenotic; SY: symplectic; U: Urostyle; UR: Urohyal; V: Vomer; VH: Ventral hypohyal; 1st DS: First dorsal spine; 2nd DS: Second dorsal spine. Scale bars indicate 10 mm.

The skeleton of *T*. *multilineatus* was divided into eight parts (cranium, vertebrae, opercular region, jaw bones, hyoid arch, mandibular region, palate-pterygoid region, and shoulder girdle region) and observed (Fig 2). The characteristics of each skeleton are described in S1 Table.

The cranium includes the occipital, otic, orbital, and ethmoid regions (Fig 2A and 2B). The occipital region, which is located on the posterior part of the cranium, consists of the supraoccipital (SO), exoccipital (EXO), and basioccipital (BO), and the otic region consists of the pterotic (PT), prootic (PR), sphenotic (SP), and epiotic (EO). The orbital region surrounding the eyes is composed of the frontal (F), prefrontal (PF), parasphenoid (PA), and pterosphenoid (PS). The ethmoid region at the front of the cranium consists of the vomer (V) and ethmoid (E).

The vertebrae are composed of 19 centrums (C) (Fig 2C). The dorsal side of the vertebral column is the neural spine (NS), while the ventral side is the hemal spine (HS). At the base of the vertebral column, there are caudal vertebrae that support the caudal fin, and the caudal vertebrae are composed of the hypural (HY), epural (EP), parhypural (PH), and urostyle (UR).

The opercular region consists of the preoperculum (POP), operculum (OP), suboperculum (SOP), and interoperculum (IOP) and is connected to the mandibular region and the cranium (Fig 2D). The function of the opercular region is to protect the face and gills. Members of the family Monacanthidae have small gill slits, and therefore their opercular region is located under the skin and immobile.

The jaw bones make up the fish jaw, and they are divided into the upper and lower jaws (Fig 2E). The premaxillary (PM) and maxillary (M) are located in the upper jaw, and the dentary (D), articular (AR), and angular (AN) are located in the lower jaw. The posterior side of the upper jaw is in contact with the V and E. The PM has three teeth on the superficial and two deep side, and the D has three teeth.

The hyoid arch is located on the facies on the medial side of the opercular region (Fig 2F). It consists of the urohyal (UR), ceratohyal (CH), epihyal (EH), interhyal (IH), dorsal hypohyal (DH), ventral hypohyal (VH), and branchiostegal rays (BR). Fibers bind CH, EH, IH, DH, and VH, and there are five BRs.

The mandibular and palato-pterygoid regions are the bones that connect to the lower jaw. The mandibular region is composed of the hyomandibular (HY), symplectic (SY), quadrate (Q), and metapterygoid (ME), and the palato-pterygoid region is composed of the palatine (P), ectopterygoid (EC), and mesopterygoid (MC) (Fig 2G). The P is in contact with the M and PM, and the SY, Q, ME, EC, and MP are combined by fibers into a single bone.

The shoulder girdle, which attaches to the posterolateral side of the cranium, consists of the cleithrum (CL), supracleithrum (SC), postcleithra (PC), scapula (SP), actinosts (AC), coracoid (CC), and pelvis (PV) (Fig 2H). Among them, the pelvis is a large bone that extends from the deep side of the clavicle to the anterior side of the anus and supports the structure for internal organs. The AC and SP are adjacent to the posterior side of the CC, and the two bones are connected to the pectoral fin.

### Genome organization and composition

**Fig 3:**
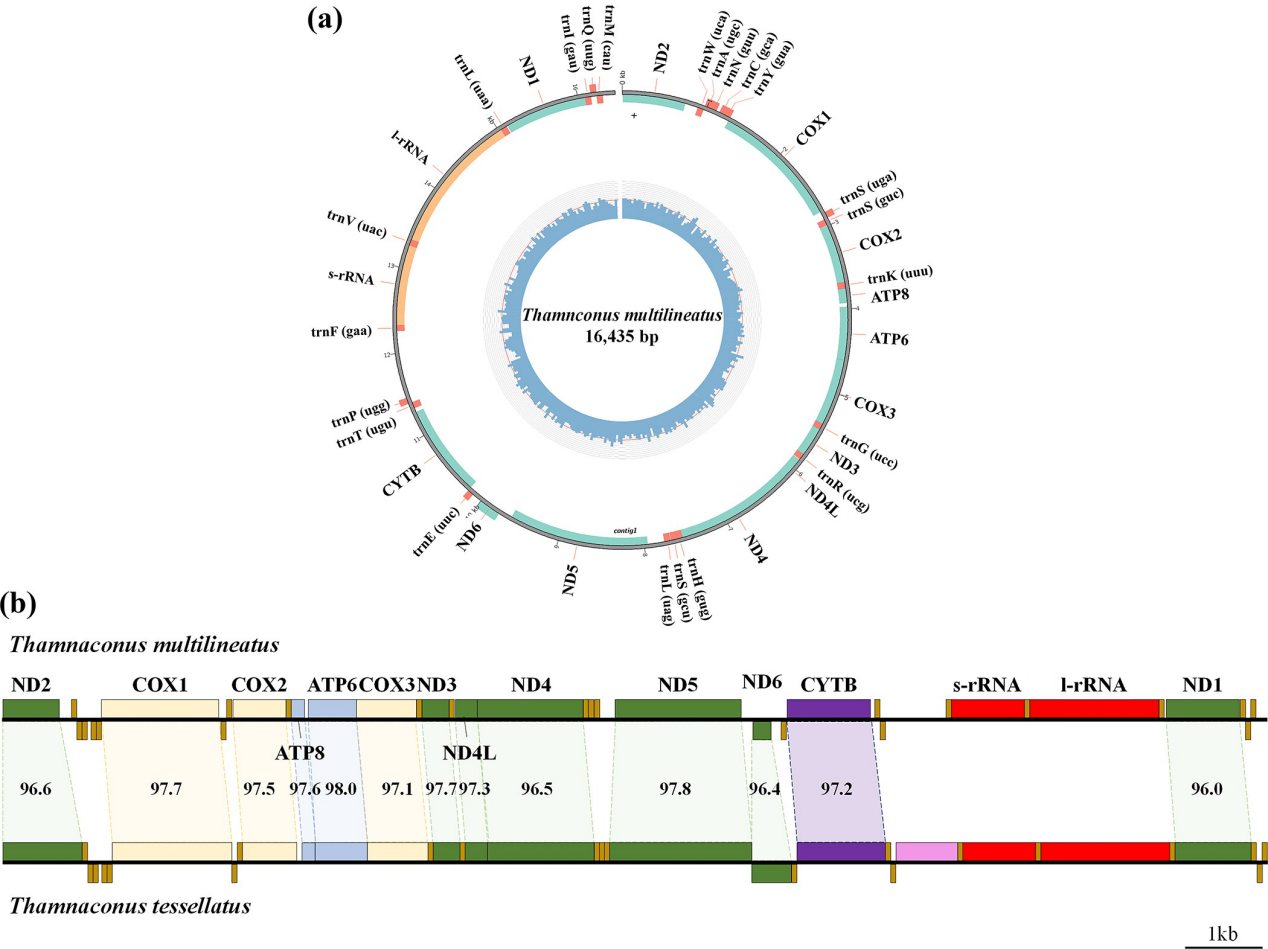
(a) The circular genome map of *Thamnaconus multilineatus*. (b) Comparison of homology for each DNA sequence between *T*. *multilineatus* and *T*. *tessellatus*.

The complete mitochondrial genome of *T*. *multilineatus* (OR402879) was sequenced to be 16,435 bp in length and contained 13 protein-coding genes (PCGs), 22 transfer RNA (tRNA) genes, and two ribosomal RNA (rRNA) genes (Fig 3A). The nucleotide composition of the whole mitogenome of *T*. *multilineatus* was as follows: (A) 27.6%, (T) 25.8%, (G) 17.2%, and (C) 29.3%. The AT content was 53.4%, and the GC content was 46.6%.

As a result of the BLASTn analysis [33], the complete mitochondrial genome of *T*. *multilineatus* showed the highest homology with *T*. *tessellatus* (KJ009561) at 97.7%. The homology range of each PCG of the two species was 96.0–98.0%, and was highest in ATP6 (98.0%), and lowest in ND1 (96.0%) (Fig 3B).

### Phylogenetic relationships

**Fig 4:**
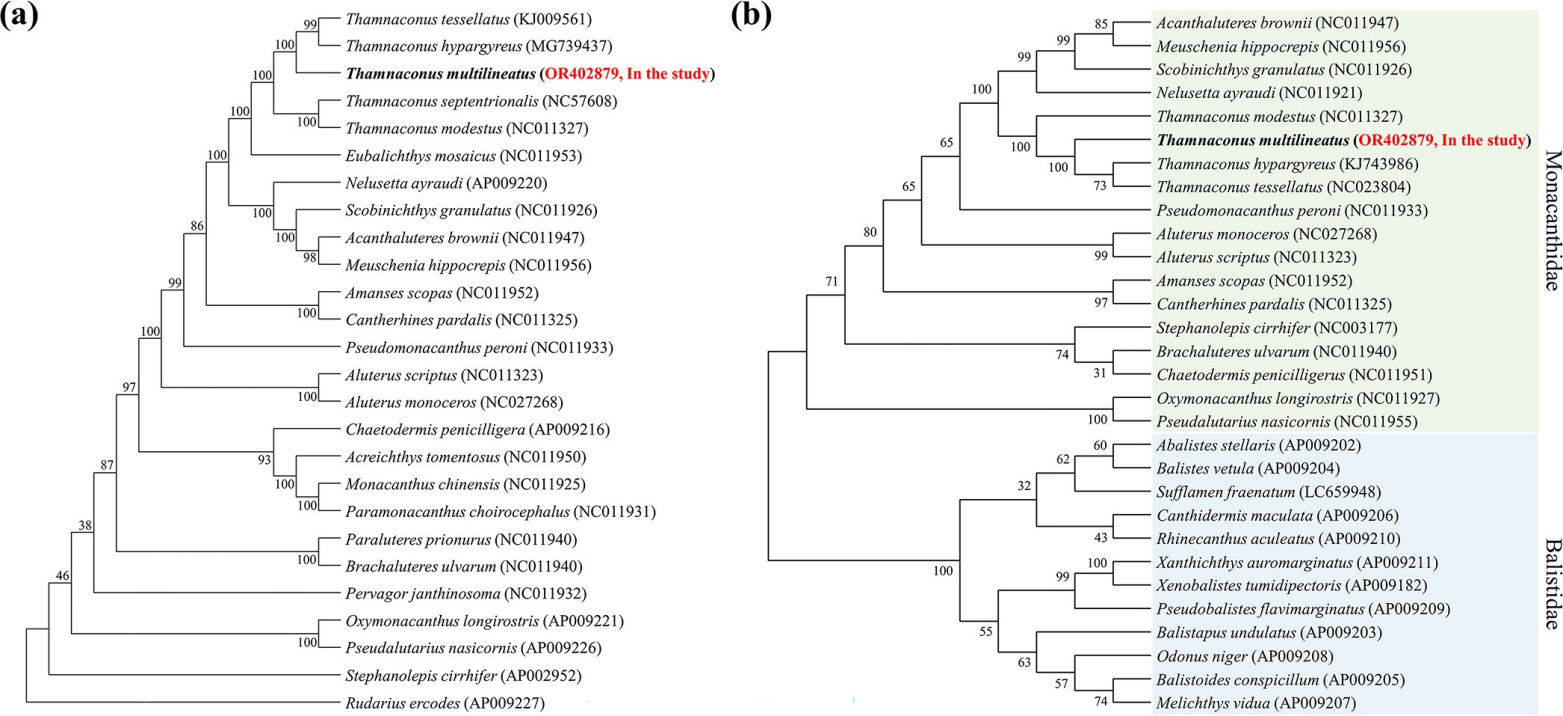
(a) The phylogenetic tree based on the complete mitochondrial DNA of the family Monacanthidae. (b) The neighbor-joining consensus tree based on mtDNA COI sequences in the family Monacanthidae and Balistidae.

The result of the phylogenetic analysis for the family Monacanthidae represented the first study of *T*. *multilineatus* within the family Monacanthidae based on the complete mitochondrial genome (Fig 4A). The *T*. *multilineatus* collected in this study formed a cluster with fish belonging to the genus *Thamnaconus* (*T*. *modestus*, *T*. *hypargyreus*, and *T*. *tessellatus*).

In the phylogenetic analysis using mitochondrial COI DNA, the families Monacanthidae and Balistidae, belonging to the suborder Balistoidei, were divided into two clusters (Fig 4B). The genetic relatedness of *T*. *multilineatus* through COI was closer in *T*. *hypargyreus* and *T*. *tessellatus* than in *T*. *modestus*.

## Discussion

Combining morphological and genetic data has historically allowed for more robust classification of organisms, enhanced our understanding of their ecology and evolution, and improved the management of these species [34]. However, species without such basic information have been classified by the IUCN Red List as Data Deficient, and 4,786 species of fish, including 24 species of the family Monacanthidae, have been registered as such. Research on Data Deficient fish species’ basic information, including their morphology and genetics, is essential for species conservation because these species are often among the most likely to be endangered due to their small numbers and rare sightings [35]. The distribution of *T*. *multilineatus*, belonging to the family Monacanthidae, has been confirmed to include Myanmar, the East China Sea, the Republic of Korea, Japan, the Philippines, Indonesia, and Fiji [4, 21, 32], but there is very limited information on their ecology, morphology, and genetic relationships.

The family Monacanthidae is diverse and has many morphologically similar species. It has been historically difficult to classify *T*. *multilineatus* between the genus *Cantherhines* and the genus *Thamnaconus* [21]. In studies comparing the morphology of *T*. *multilineatus* to that of *C*. *dumerilii* and *C*. *pardalis*, *T*. *multilineatus* and *C*. *pardalis* were found to have no spines on the caudal peduncle, whereas *C*. *dumerilii* had two pairs of spines [4, 36]. In addition, there are morphological differences among *T*. *multilineatus*, *T*. *hypargyreus*, and *T*. *tessellatus* in body surface patterns. While brown vertical stripes are scattered across the lower 2/3^rds^ of the eyes of *T*. *multilineatus*, *T*. *hypargyreus* and *T*. *tessellatus* have a brown dot pattern on the body. Compared to that of other members of the genus *Thamnaconus*, the skeleton of *T*. *multilineatus* differed in the number of branchiostegal rays and the shape of the suboperculum; however, classification and phylogenetic analysis using only these morphological traits are difficult [37, 38]. Character adaptation and convergence problems, which sometimes may obscure the morphological analysis, are reduced if DNA sequences of marker genes are used [39].

The mtDNA genome has many special features such as its maternal inheritance and high mutation rate which have made it attractive to scientists [40]. In addition, the distribution of different nucleotide variations across global populations is not uniform, allowing the geographical ancestry of biological evidence to be inferred using mtDNA and making it a valuable tool for species identification [41]. Twenty-five complete mtDNA genomes belonging to fish of the family Monacanthidae have been registered in the NCBI (https://www.ncbi.nlm.nih.gov/), but there is no information on the complete mtDNA of *T*. *multilineatus*. The complete mtDNA of *T*. *multilineatus* was analyzed in this study for the first time. It consisted of 37 genes (16,435 bp), and was highly homologous with *T*. *tessellatus* at 97.7%. The cytochrome c oxidase subunit I (COXI) and cytochrome b genes have been used in numerous studies of phylogenetic relationships within organisms, and they are the genes for which the most sequences from different species are available [42].

In this study, we provided crucial information such as morphology and complete mtDNA to be used for the resource management and preservation of *T*. *multilineatus*. By synthesizing previous studies on *T*. *multilineatus*, we confirmed that the species is distributed in the Indo-West Pacific; still, we could not evaluate population size because of insufficient data. To preserve marine biodiversity and maintain viable ecosystems, studies need to continuously accumulate information for species identification. In particular, the morphological and genetic results obtained through this study can be used in related research, such as exploring *T*. *multilineatus* ecology, evolution, and adaptation to environmental changes.

## Supporting information

S1 Table Characteristics of the skeleton of *Thamnaconus multilineatus*. (DOCX)
