# Supplementary material for: Morphological and genetic analysis for the diversity conservation of rare species, Thamnaconus multilineatus (Tetraodontiformes: Monacanthidae)
Source: PLoS One. 2024 Feb 29;19(2):e0292916. doi: 10.1371/journal.pone.0292916 (PMC10903791; doi:10.1371/journal.pone.0292916)
Supplement: S1 Table — (DOCX) [file pone.0292916.s001.docx]

S1 Table. Characteristics of the skeleton of *Thamnaconus multilineatus*

| Skeletal region | Skeleton | Characteristics |
| --- | --- | --- |
| Cranium | Supraoccipital | It widens towards the rear, and the back is relatively flat. It is covered by the base of the first dorsal fin, connected to the back by cartilage, and not exposed. |
|  | Exoccipital | Through cartilage, it articulates dorsally with the epiotic, laterally with the pterotic, and ventromedially with the basioccipital. Towards the back, it is firmly attached by fibrous tissue and interlocks with the elongated first vertebra on the lateral side. |
|  | Basioccipital | It is a short, anteriorly extended cylindrical form. Towards the dorsal side, it articulates with the exoccipitals, on the anterior ventrolateral side with the prootic, and on the anterior inner side with the upper posterior part of the parasphenoid. The concave posterior part is connected to the first vertebra and fibrous tissue. The ventromedial surface on the ventral side is concave. |
|  | Pterotic | On the dorsal side, it connects with the epiotic through cartilage articulation, and towards the posterior side, it connects with the exoccipital. On the ventromedial surface, it interfaces with the basioccipital and parasphenoid, while on the anterior outer side, it contacts the sphenotic, and on the lateral side, it touches the posttemporal. The ventromedial portion on the ventral side is deeply embedded, and fibrous tissue anchors the hyomandibular in this area. |
|  | Prootic | The inner side of the prootic is in contact with the outer side of the parasphenoid, and the anterior outer side contacts the sphenotic, while the posterior side interfaces with the pterotic. The inner edge forms the lateral wall of the myodome, and the front end is connected to the muscle that touches the subocular. |
|  | Sphenotic | It combines the pterosphenoid, prootic, and pterotic on the anterior inner side, interfaces with the frontal on the anterior outer side, and on the posterior side, it touches the epiotic. |
|  | Epiotic | It is extensively interlocked with the basal pterygiophore of the first dorsal fin, and its dorsal edge aligns with the center of the cranium, making contact with the opposite skeleton. It is connected by interfacing with the posterior edge of the supraoccipital toward the front and linked to the pterotic and exoccipital on the ventral side. |
|  | Frontal | The posterior part of the frontal is the widest, becoming thinner as it extends towards the front. The lateral edges above the orbit are thin but become thicker as they go upwards. It interfaces with the flat posterior area of the supraoccipital and is connected to the sphenotic towards the rear. Towards the front, it connects with the ethmoid, and on the sides, it is linked with the prefrontal. |
|  | Prefrontal | It extends dorsolaterally and is connected to the frontal by cartilage. |
|  | Parasphenoid | It has an elongated form that extends towards the ventral side until the front of the orbit. The posterior part of the parasphenoid has a pair of short and slightly split protrusions that connect to the prootic. The anterior dorsal surface is concave and connected to the ethmoid through fibrous tissue. The anterior end is deep and concave to accommodate the posterior part of the vomer. |
|  | Pterosphenoid | It is filled with cartilage along the edges of the pterosphenoid and is connected to the frontal, sphenotic, and prootic through fibrous tissue. |
|  | Vomer | It is a vertically extended bone that expands on the lateral side. It is connected to the parasphenoid by fibrous tissue towards the back and supports the upper jaw towards the front. |
|  | Ethmoid | It has a long and deep form. It extends laterally along the dorsal side, but on the ventral side, there is a deep and flat plate connecting to the parasphenoid's concave part. The ventral edge of the ethmoid is connected to the vomer anteriorly, and on the posterior side, it overlaps with the frontal. The front end of the ethmoid extends on both sides and makes contact with the palatine and premaxillary. |
| Opercular region | Preoperculum | It has a triangular shape with a concave central part. The posterior side is slightly flat, while the anterior is relatively thin and thick. |
|  | Operculum | It is generally thin and flat but thickens towards the upper side, forming a rounded shaft with an articular facet. This facet articulates through fibrous tissue with the posterior edge of the dorsal part of the hyomandibular. The suboperculum covers it on the lower side and articulates through fibrous tissue. |
|  | Suboperculum | It is very thin and flat and overlaps with the operculum through fibrous tissue. |
|  | Interoperculum | It is a thin, elongated, rod-like skeletal structure extending from the interhyal to the preoperculum. A strong ligament firmly attaches the front end to the lower jaw. |
| Jaw bone | Premaxillary | It is medially curved plate, wider along its lower edge than its upper edge, and articulates posteriorly with the ethmoid and vomer. The maxillary is firmly attached to the upper surface of the premaxillary. Five teeth are arranged in two rows, with three on the outer row and two on the inner row. |
|  | Maxillary | The ventral portion is ruggedly shaped, tightly adjacent to the premaxillary. The upper posterior part forms joints with the ethmoid, vomer, and palatine through fibrous tissue. |
|  | Dentary | Towards the back, it is broad but concaves inward to accommodate the articular and angular. On the lateral side, it connects with the inner surface of the premaxillary bone and bears a row of three teeth. |
|  | Articular | The posterior side is broad and connected to the quadrate's anterior part through fibrous tissue. The concave posteromedial surface articulates with the dentary, and the posterior edge is interlocked with the angular. |
|  | Angular | A small wedge-shaped bone interlocked dorsally with the articular bone and connected anteriorly with the dentary bone. Posteriorly, the angular bone is connected with the interoperculum through ligaments. |
| Hyoid arch | Urohyal | It has a beak-like shape and is located at the central part of the body between the two ventral hypohyals on both sides. |
|  | Ceratohyal | It is a flat skeletal structure with a relatively wider posterior than the anterior. It forms joints through cartilage with the dorsal and ventral hypohyal, as well as the epihyal. The first branchiostegal ray is positioned in the middle of the lateral side, and the four branchiostegal rays are situated over the epihyal towards the rear. |
|  | Epihyal | It is similar in size to each hypohyal. It articulates dorsally with the interhyal and interoperculum, and ventrally with the ceratohyal, forming joints through fibrous tissue. |
|  | Interhyal | It is a short, rod-shaped bone, with the epihyal connected on the ventral side and the symplectic on the dorsal side through fibrous tissue. |
|  | Hypohyal | The hypohyal is divided into dorsal and ventral hypohyal, with similar sizes. Both hypohyals form joints through cartilage and articulate with the ceratohyal towards the posterior side. On the inner side, they anchor the urohyal. |
|  | Branchiostegal rays | There are five branchiostegal rays. The first branchiostegal ray is articulated with the ceratohyal, and four branchiostegal rays are arranged on the lateral sides of the ceratohyal and epihyal. The first branchiostegal ray is wider than the other rays, and the other rays are thin, elongated, and rod-shaped. |
| Mandibular region | Hyomandibular | The anterior part is thin, and it thickens posteriorly. The lateral side is connected to the preoperculum through fibrous tissue. The front ventral side is connected to the metapterygoid, symplectic, interhyal, and preoperculum. |
|  | Symplectic | Its flat shaft-like form forms one bone with the metapterygoid towards the back and the quadrate towards the front. It is connected to the preoperculum through fibrous tissue on the ventral side and is positioned between the interhyal and metapterygoid. |
|  | Quadrate | The rear part is the widest, and the front part is elongated to connect with the lower jaw joint. The posterior part of the quadrate is split along the edge to combine with the front of the symplectic, and the posterior side connects with the mesopterygoid and metapterygoid. |
|  | Metapterygoid | It has a bent and flat plate shape along the dorsal edge. The front is connected to the quadrate and is linked anterodorsally to the mesopterygoid and anteroventrally to the symplectic. Along the ventral edge, it connects the interhyal. |
| Palato-pterygoid region | Palatine | It is a skeletal structure of short form, connected to the ectopterygoid by fibrous tissue. Towards the front, it is connected to the maxillary and extends up to the vomer. |
|  | Ectopterygoid | It is triangular in shape, long, and curved. On the ventral side, it articulates with the quadrate, and on the posterior side, it forms a joint with the mesopterygoid. |
|  | Mesopterygoid | It is connected to the ectopterygoid towards the front and the metapterygoid towards the back. |
| Shoulder girdle | Cleithrum | The anterior part has a rounded and elongated depression. The supracleithrum and postcleithrum are in contact through the fibrous tissue above. Along the posterior edge, the scapula and coracoid overlap. Between the two cleithrum on the inner side, it is strongly attached to the front part of the pelvis. |
|  | Supracleithrum | It is a straight skeletal structure that extends vertically along the body axis. The inner side is adjacent to the upper part of the cleithrum through fibrous tissue, and the dorsal side is anchored to the posttemporal bone. |
|  | Postcleithrum | The postcleithrum is a long, flat, and robust bone. The dorsal end is connected to the posterior side of the cleithrum through fibrous tissue. It slopes obliquely towards the pelvis. |
|  | Scapula | It surrounds the scapular foramen. It is anteriorly connected to the cleithrum through fibrous tissue and ventrally linked to the coracoid. |
|  | Actinosts | It consists of four bones. The first actinost is small, and its size increases slightly as it moves downwards. Except for the first coracoid supported by the scapula, the rest of the bones support the pectoral fin. |
|  | Coracoid | The dorsal side is broad and tapers down towards the lower side. A thin spine is on the posterior side of the ventral part. It is connected to the posterior part of the cleithrum through fibrous tissue. On the posterior side, it is joined with the scapula and supports the actinost. |
|  | Pelvis | It is a long, thick skeleton extending from the cleithrum to the anterior anus. The lateral side is concave to allow muscle attachment. The front is fixed by cartilage, but the rear can move up and down. The posterior end is divided into two branches, protruding outward with small spines underneath. |
